# Left Ventricle Outflow Tract Velocity-Time Index and Right Ventricle to Left Ventricle Ratio as Predictors for in Hospital Outcome in Intermediate-Risk Pulmonary Embolism

**DOI:** 10.3390/diagnostics12051226

**Published:** 2022-05-13

**Authors:** Elena Emilia Babes, Manuela Stoicescu, Simona Gabriela Bungau, Diana Uivarosan, Delia Mirela Tit, Mirela Marioara Toma, Alexa Florina Bungau, Cristiana Bustea

**Affiliations:** 1 Department of Medical Disciplines, Faculty of Medicine and Pharmacy, University of Oradea, 410073 Oradea, Romania; babes.emilia@gmail.com; 2 Doctoral School of Biological and Biomedical Sciences, University of Oradea, 410087 Oradea, Romania; mirela_tit@yahoo.com (D.M.T.); mire.toma@yahoo.com (M.M.T.); 3 Department of Pharmacy, Faculty of Medicine and Pharmacy, University of Oradea, 410028 Oradea, Romania; 4 Department of Preclinical Disciplines, Faculty of Medicine and Pharmacy of Oradea, University of Oradea, 410073 Oradea, Romania; diana.uivarosan@gmail.com; 5 Medicine Program of Study, Faculty of Medicine and Pharmacy, University of Oradea, 410073 Oradea, Romania; pradaalexaflorina@gmail.com (A.F.B.); cristianabustea@yahoo.com (C.B.)

**Keywords:** pulmonary embolism, intermediate risk, left ventricular outflow tract, velocity time integral, right ventricle dysfunction

## Abstract

Accurate estimation of risk with both imaging and biochemical parameters in intermediate risk pulmonary embolism (PE) remains challenging. The aim of the study was to evaluate echocardiographic parameters that reflect right and left heart hemodynamic as predictors of adverse events in intermediate risk PE. This was a retrospective observational study on patients with computed tomography pulmonary angiography diagnosis of PE admitted at Cardiology department of the Clinical Emergency Hospital of Oradea, Romania between January 2018—December 2021. Echocardiographic parameters obtained at admission were studied as predictors of in hospital adverse events. The following adverse outcomes were registered: death, resuscitated cardiac arrest, hemodynamic deterioration and need of rescue thrombolysis. An adverse outcome was present in 50 patients (12.62%). PE related death was registered in 17 patients (4.3%), resuscitated cardiac arrest occurred in 6 patients (1.51%). Another 20 patients (5.05%) required escalation of therapy with thrombolysis and 7 (1.76%) patients developed haemodynamic instability. Echocardiographic independent predictors for in hospital adverse outcome were RV/LV ≥ 1 (HR = 3.599, 95% CI 1.378–9.400, *p* = 0.009) and VTI ≤ 15 mm (HR = 11.711, 95% CI 4.336–31.633, *p* < 0.001). The receiver operator curve renders an area under curve for LVOT VTI ≤ 15 mm of 0.792 (95% CI 0.719–0.864, *p* < 0.001) and for a RV/LV ≥ 1 of 0.746 (95% CI 0.671–0.821, *p* < 0.001). A combined criterion (LVOT VTI ≤ 15 and RV/LV ≥ 1) showed a positive predictive value of 75% and a negative predictive value of 95% regarding in hospital adverse outcomes. Low LVOT VTI and increased RV/LV are useful for identifying normotensive patients with PE at risk for short term adverse outcomes. Combining an LVOT VTI ≤ 15 cm with a RV/LV ≥ 1 can identify with increased accuracy PE patients with impending risk of clinical deterioration.

## 1. Introduction

Pulmonary embolism (PE) is a major cause of morbidity and mortality. The annual incidence rate of PE is increasing over time as revealed in longitudinal studies [1,2,3,4]. The rising incidence is correlated with increasing of life expectancy, a higher incidence of risk factors for PE and the large availability of more sensitive imaging diagnostic techniques [5]. The annual incidence of PE in Europe is 115/100,000 population [6]. Acute PE is the third leading cause of death globally, although most of the patients have a low mortality rate and can be treated by anticoagulation alone [7]. Around 10–30% of deaths occur in the first month after presentation [8]. Mortality rates at 30 day and 1-year are 3.9 and 12.9% and increase with the severity of PE [9]. After an episode of PE patients may experience a marked impairment of quality of life due to development of chronic thromboembolic pulmonary hypertension [10].

There are several risk factors for venous thromboembolism that result from an interaction between the individual patient’s risk factors (usually persistent) and setting-related (usually transitory) risk factors. Age, personal history of venous thromboembolism, cancer, comorbidities such as heart failure or respiratory diseases, coagulation disorders, oral contraception and pregnancy are described as predisposing factors for PE. Surgery, orthopedic procedures (lower-limb fractures and joint replacements) and major trauma are associated with increased risk of PE. Common risk factors are shared by PE and atherosclerosis, respectively hypertension, diabetes, hypercholesterolemia, obesity, and smoking. Atherosclerosis and arterial disease may be related to PE by increased platelet activation and coagulation pathway [6].

The clinical presentation of patients with PE is highly variable with a spectrum from critically ill to stable patients. Guidelines differentiate between high-risk, intermediate, and low risk patients. Risk stratification of patients will influence therapeutic management. High risk (massive) PE is associated with hemodynamic instability, respectively obstructive shock, persistent arterial hypotension refractory to treatment, or cardiac arrest. In these patients right ventricular dysfunction (RVD) should be confirmed on echocardiography, to demonstrate that hemodynamic impairment is secondary to PE and thrombolysis is recommended [6]. Low risk PE patients are hemodynamic stable with no signs of RVD on echocardiography or laboratory markers of injury and for them anticoagulation therapy will suffice.

Intermediate-high risk PE encompasses a group of patients with normal blood pressure, signs of right ventricular dysfunction (RVD) on echocardiography or computed tomography pulmonary angiography (CTPA) and elevated cardiac biomarkers (brain natriuretic peptide, troponin). The low-intermediate risk patients have either imaging evidence of RVD or elevated cardiac biomarkers but not both or are classified as being not low-risk by clinical prediction tools. These intermediate risk patients are apparently hemodynamically stable, but up to 10% of them will progress to massive PE with high mortality. Short term mortality rates vary between 2–10% in intermediate-risk patients [5]. There are still controversies regarding the best approach in the management of these patients. Thrombolysis in hemodynamically stable patients with intermediate-high risk PE continues to be an area of debate. Anticoagulant therapy will cause a passive reduction of thrombus size while thrombolytic agents will determine a more rapid clot resolution, improvement of cardiac and respiratory function and symptoms resolution. Although thrombolysis is reasonable in patients with massive acute PE, routine thrombolysis is not recommended in patients with intermediate risk PE due to high risk of bleeding complications. They should be closely monitored over the first days because there is a risk of hemodynamic instability [6]. These patients with intermediate-high risk PE do not necessarily need thrombolysis but sometimes anticoagulant therapy is not sufficient to prevent haemodynamic decompensation.

It is a challenge to identify those patients with intermediate risk who will experience complications and will require escalation of therapy. Rescue systemic thrombolysis, percutaneous catheter-directed thrombolysis or surgical embolectomy are treatment options in patients who become unstable [11,12]. The aggressiveness of therapy must match the severity of disease. The decision of thrombolysis requires careful consideration of the risks and benefits involved and appropriate stratification of risk. The risk of hemodynamic deterioration and death may be difficult to assess and already developed risk scores as Pulmonary Embolism Severity Index (PESI) or BOVA perform better in identification of low-risk patients and can underestimate patient severity.

Echocardiography plays an important role in PE, being recommended for risk estimation by current ESC guidelines [6]. Detecting RVD by imaging performs better than risk stratification scores as PESI or BOVA in predicting poor prognosis in patients with intermediate-risk PE [13]. Even in patients evaluated as being at low risk on clinical models the detection of RVD at echocardiography or CTPA especially associated with increased troponin or brain natriuretic peptide/N-terminal pro b-type natriuretic peptide (BNP/NT-proBNP) is correlated with early death risk [14]. A large number of studies evaluated various echocardiographic elements of RVD as predictors of increased mortality in initially stable patients [15,16,17,18,19,20,21,22].

Acute RV pressure overload and RVD can be detected with echocardiography although the geometry of the RV is complex, and evaluation of RV size and dysfunction is quite challenging. Various echocardiographic parameters were reported for describing RV in PE such as: RV enlargement, RV hypokinesis, flattening or bowing of the interventricular septum, an elevated velocity of tricuspid valve regurgitation, tricuspid annular plane systolic excursion (TAPSE), the combination of a pulmonary ejection time <60 ms with a peak systolic tricuspid valve gradients <60 mmHg and were correlated with outcome in acute PE [5,22,23]. Controversial results are persisting regarding correlation between various echocardiographic parameters of RVD and adverse short-term outcomes in patients with PE. RVD diagnosed by different echo parameters is associated with mortality rates from 4.3% to 16.4% [17,22]. Several studies revealed a modest increased in mortality associated with various parameters of RVD [16,17,18,24,25], while in other studies this was not confirmed [26,27]. A meta-analysis performed by Coutance et al. revealed that although RVD is correlated with high-risk short-term mortality in PE the positive predictive value for death is low [22].

Recently, low left ventricular outflow tract velocity-time integral (LVOT VTI), a marker of LV stroke volume, was demonstrated to predict adverse outcomes in patients with acute PE [27]. LVOT VTI is a reliable surrogate for cardiac output in the absence of LVOT abnormalities, is less prone to error than the estimation of LVOT area and can outperform ejection fraction [28,29].

The aim of this research was to evaluate echocardiographic parameters that reflect RVD and left ventricular underfilling, including RV/LV ratio and LVOT VTI as predictors of in hospital adverse outcome in initially stable patients with intermediate risk PE. To our knowledge this is the first study that studied the value of a combined criteria of LVOT VTI ≤ 15 cm and an RV/LV ratio ≥ 1 in risk stratification of patients with PE.

## 2. Materials and Methods

### 2.1. Study Design

A retrospective observational study was performed on 708 patients consecutively admitted with the diagnosis of acute PE in the Cardiology Department of the Clinical County Emergency Hospital of Oradea Romania between January 2018 and December 2021. Inclusion criteria was the diagnosis of acute PE confirmed by contrast enhanced computed tomographic pulmonary angiography (CTPA). Patients presenting with acute PE were classified according to European Society of Cardiology criteria for PE severity. Only patients classified in the intermediate risk class, hemodynamically stable on admission, with systolic blood pressure (BP) of at least 90 mmHg without hemodynamic support were included in the study. Exclusion criteria were as follows: patients with high LVOT velocities due to moderate-severe aortic regurgitation where the estimation of LVOT VTI is not reliable due to overestimation of forward stroke volume, patients with hypertrophic obstructive cardiomyopathy, sepsis, and hypovolemia with dynamic obstruction of LVOT, patients with LVOT VTI with insufficient quality to trace, patients with incomplete echocardiographic data, patients known with chronic pulmonary hypertension.

All patients were treated according to ESC guidelines. They initially received standard anticoagulation therapy most of them with intravenous unfractionated heparin or subcutaneous low-molecular weight heparin. Thrombolytic treatment, respectively systemic thrombolysis with t-PA was administered only when hemodynamic deterioration occurred at the indication of the attending physician or on call physician.

The following adverse events were recorded during hospitalization period: death or resuscitated cardiac arrest, development of hemodynamic instability: cardiogenic shock or need of hemodynamic support, need of systemic rescue thrombolysis. All the patients included in the study had echocardiographic examinations performed in the first 24–48 h after admission. Echocardiographic data were compared between groups of patients with and without adverse events, and those parameters that differed significantly between groups were evaluated regarding their predictive power in multivariate analysis.

The entire study was conducted respecting the World Medical Association Declaration of Helsinki (Ethical Principles for Medical Research Involving Human Subjects) and was approved by the Ethics Committee of the Clinical County Emergency Hospital of Oradea, Bihor County, Romania (decision no 9411/08.04.2021). Each patient included in the study signed the informed consent form.

### 2.2. Methodology

The diagnosis of PE was defined on CTPA as the presence of a thrombo-embolus in at least one segmental pulmonary artery. Patients with normal BP on admission, PESI III-V or s PESI ≥ 1 in the intermediate risk class were included in the study. The presence of signs of RVD on echocardiography or computed CTPA and elevated cardiac biomarkers categorized patients according to ESC guidelines [6] in the intermediate high- risk category. The low-intermediate risk patients had either imaging evidence of RVD or elevated cardiac biomarkers but not both or were classified as being not low-risk by clinical prediction tools.

Data were obtained from patient’s medical records and from hospital informatic system. Demographic data, medical history, clinical and paraclinical data, risk factors were recorded. Plasma cardiac biomarkers as N-Terminal Pro-B-Type Natriuretic Peptide (NT proBNP) and high sensitive (hs)-troponin were determined on admission. NT-proBNP was determined by using a path fast cardiac biomarker analyzer manufactured by LSI Medience Corporation, a bench-top chemiluminescent immunoassay analyzer providing affordable, core-lab quality results from whole blood. Hs-troponin was determined with Alinity High Sensitive Troponin-I assay (Abbott Diagnostics).

A set of echocardiographic parameters of patients who presented with PE were recorded in the first 24–48 h after admission on a Siemens Acuson X 300 (Siemens Medical Solutions USA, Inc., Seoul, Korea) or on a Philips CX50 POC (Philips Healthcare, Makati, Philippines) system. The selected studied parameters are part of a standard echocardiography examination, are easy to determine, without important interobserver variability and do not require special technical equipment or skills.

The following parameters that reflect right heart changes in PE were recorded: dilatation of the right ventricle (RV) was assessed in apical four chamber view with by measuring LV and RV end-diastolic diameters at the level of mitral and tricuspid valve tips and RV/LV ratio was calculated (RV/LV ratio > 1 was considered elevated); the presence of McConnel sign: hypokinesia of RV free wall with good apical contractions; tricuspid annulus plane systolic excursion (TAPSE) a parameter that reflects RV contractions was measured in M-mode with a normal value >16 mm; pulmonary artery systolic pressure (PSAP) was determined from Doppler derived tricuspid regurgitation velocity using the simplified Bernoulli method; the presence of a paradoxical or flattening septal motion a classic 2D sign indicating an increased pressure in the RV was assessed by visual estimation; the diameter of inferior vena cava was assessed at late expiration. Echocardiographic parameters that reflect the left heart can reveal reduced left heart filling with diminished cardiac output in PE. LVOT VTI was obtained by tracing the envelope of the Doppler spectrum of LVOT systolic flow from the apical five- or three-chamber view using pulsed-wave Doppler, with the sample volume placed within the LVOT, approximately at 1 cm below the aortic valve. The pulsed-wave Doppler sample volume was placed parallel to the subaortic flow for obtaining an optimal VTI with minimal spectral broadening [30]. In patients with atrial fibrillation an average of 5 VTI s was calculated due to beat to beat VTI variability. An LVOT VTI ≤ 15 cm is commonly accepted as low, and this value was used also in our study [31]. Ejection fraction was calculated using bi-plane method of Simpson from apical two and four chambers’ views.

PE related death was confirmed at autopsy or occurred shortly after a clinically severe PE or in the absence of an alternative diagnosis. Cardiac arrest was defined as the need for cardiopulmonary resuscitation. Development of hemodynamic instability was defined as the occurrence of cardiogenic shock defined as sustained reduction of systolic blood pressure < 90 mmHg for at least 15 min or requiring pressor support, or a systolic BP drop ≥ 40 mmHg for >15 min, not caused by new-onset arrhythmia, hypovolemia, or sepsis. Need of systemic rescue thrombolysis was defined as escalation of therapy due to hemodynamic instability and was established at the indication of the attending physician or on call physician.

### 2.3. Statistical Analysis

Data were analyzed using SPSS statistical package Version 25 [32] and MedCalc statistical software version 19.4 (MedCalc Software, Ostend, Belgium). Results are presented as mean ± SD for continuous variable and frequencies and percentage for categorical variables. Intergroup comparison was made using independent sample t test for continuous variables and Kruskal Wallis test for categorical variable. A value of *p* < 0.05 was considered statistically significant. For those parameters that were significantly different between groups with and without adverse outcome a multiple regression analysis was performed to determine the value of each parameter as independent predictor for an adverse event. ROC curve analysis was performed for independent predictors of adverse events with determination of AUC (area under curve) to evaluate the accuracy or the performance of these parameters in predicting mortality or adverse outcome. Sensitivity, specificity, positive and negative predictive value were calculated.

The sample size was calculated using the on-line software Open Source Epidemiologic Statistics for Public Health (OpenEpi) version 3.01 [33].

## 3. Results

**Figure 1:**
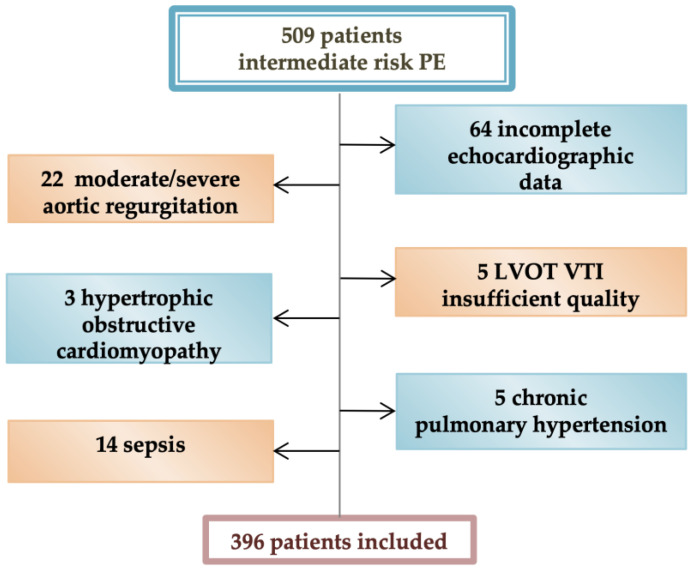
Study flow diagram.

A number of 708 patients were consecutively admitted with the diagnosis of acute PE. According to ESC guidelines classification, 509 normotensive patients were included in the intermediate class risk at admission; 396 of them were included in the final evaluation after applying the exclusion criteria (Figure 1).

Adverse outcome (including in hospital death, cardiac arrest, development of cardiogenic shock or sustained hypotension, need of rescue thrombolysis) was present in 50 patients (12.62%). PE related death was registered in 17 patients (4.3%) and resuscitated cardiac arrest occurred in 6 patients (1.51%). Another 20 patients (5.05%) required escalation of therapy with thrombolysis and 7 (1.76%) patients developed hemodynamic instability and received intravenous inotropes. Echocardiographic parameters including RV/LV ratio and LVOT VTI to predict PE-related adverse events, including mortality, cardiac arrest, hemodynamic instability, need of rescue thrombolysis during hospitalization were directly compared in 396 normotensive patients with acute PE.

### 3.1. Predictors of in Hospital Adverse Outcomes: Death, Resuscitated Cardiac Arrest, Hemodynamic Instability and Need of Reperfusion Therapy

Demographic, clinical characteristics and biomarkers values of patients that developed adverse outcomes during hospitalization compared to those with favorable evolution are recorded in Table 1.

Unfavorable outcome was significantly more frequent in in patients with previous PE/DVT. Patients with adverse short-term events had significantly higher hs-troponin level at admission compared to those with favorable outcome. Echocardiographic parameters were measured in the first 24 h after admission in 327 (82.58%) patients and in the first 48 h for the rest of 69 (17.42%) of patients and are revealed in Table 2.

**Figure 2:**
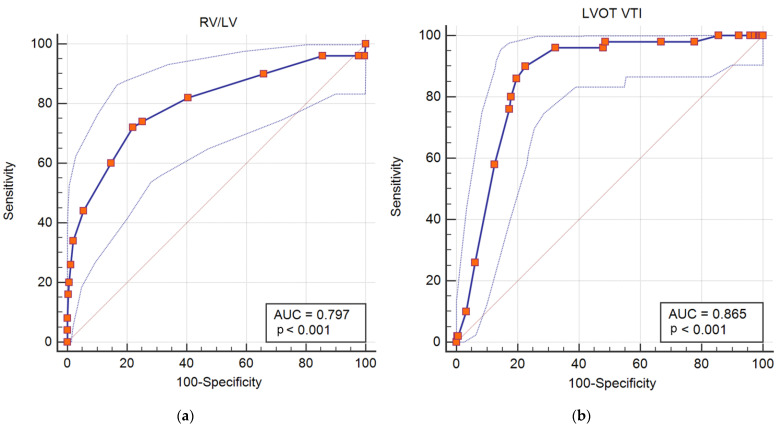
ROC curve for (**a**) RV/LV as predictor of adverse events in intermediate risk PE; (**b**) LVOT VTI as predictor of adverse outcome in intermediate risk PE.

RVD was more pronounced in patients with unfavorable outcome. A higher RV to LV ratio and a lower TAPSE were measured compared to those with uncomplicated evolution. LVOT VTI was significantly reduced in patients with adverse outcome. In multiple regression analysis independent predictor for in hospital adverse outcome remained high-sensitive troponin level (HR = 1.001, 95% CI 1.000–1.002, *p* = 0.04), RV to LV ratio ≥ 1 (HR = 3.599, 95% CI 1.378–9.400, *p* = 0.009) and LVOT VTI ≤ 15 cm (HR = 11.711, 95% CI 4.336–31.633, *p* < 0.001). The AUC for RV to LV ratio as predictor of adverse outcome was 0.797 (95% CI 0.720–0.874, *p* < 0.001) (Figure 2a). The AUC for LVOT VTI as predictor of adverse outcome was 0.865 (95% CI 0.821–0.910, *p* < 0.001) (Figure 2b).

**Figure 3:**
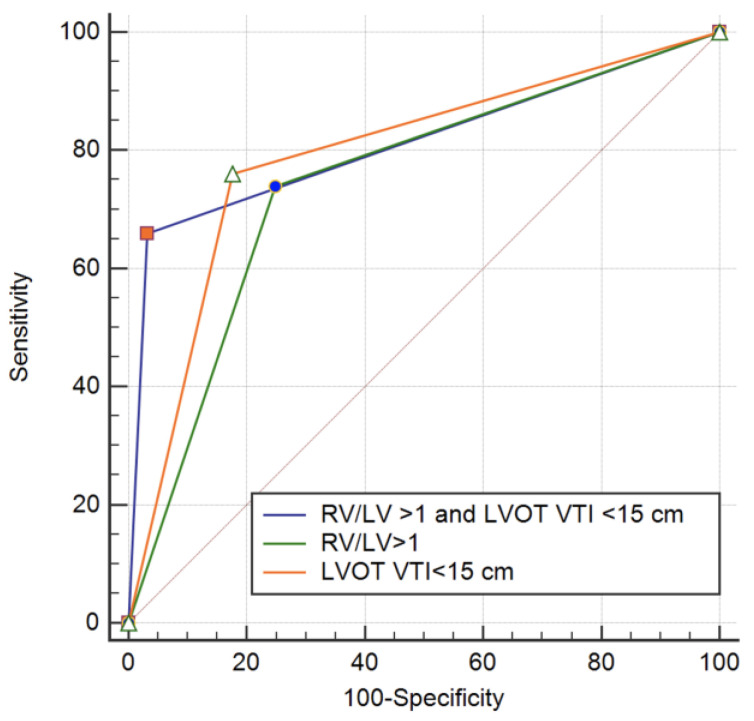
ROC curves for adverse event prediction in intermediate risk PE.

The AUC for an RV to LV ratio ≥ 1 was 0.746 (95% CI 0.671–0.821, *p* < 0.001) and for LVOT VTI ≤ 15 cm was 0.792 (95% CI 0.719–0.864, *p* < 0.001) (Figure 3). LVOT VTI ≤ 15 cm had a sensitivity of 76% and a specificity of 82.4% in predicting adverse in hospital outcome. The positive predictive value was 38% and the negative predictive value was 95% for an LVOT VTI ≤ 15 cm.

RV to LV ratio ≥ 1 is a predictor of adverse outcome with a sensitivity of 74%, and a specificity of 75.1%. The positive predictive value for a RV to LV ratio ≥ 1 was 30% and the negative predictive value was 95%. A combined criteria of RV/LV ratio ≥ 1 and an LVOT VTI ≤ 15 mm was present in 44 patients (11.1%). From these patients 33 (66%) had an unfavorable in hospital outcome. The ROC analysis for a combined parameter showed an AUC of 0.814 (95% CI 0.733–0.895, *p* = 0.041) (Figure 3). A combined parameter had a sensitivity of 66% and a specificity of 96% in predicting an adverse outcome. The positive predictive value of a combined parameter is significantly increased to 75% with a negative predictive value of 95%.

### 3.2. Predictors of in Hospital Death and Resuscitated Cardiac Arrest

A number of 23 (5.8%) patients experienced death or resuscitated cardiac arrest during hospitalization in intermediate-risk patients. Demographic, clinical and laboratory characteristics are revealed in Table 3.

Patients that experienced cardiac arrest were more commonly female, had lower systolic BP and higher NT-pro BNP and troponin levels at admission. Echocardiographic parameters were compared between patients with and without in hospital cardiac arrest and are revealed in Table 4.

An RV/LV ratio ≥ 1 and a low LVOT VTI were significantly more common patients within hospital death or resuscitated cardiac arrest. In multiple regression analysis only a LVOT VTI ≤ 15 cm remained independent predictor for death and resuscitated cardiac arrest HR = 8.117 (95% CI 1.941–33.946, *p* = 0.004).

### 3.3. Predictors of in Hospital Complications: Hemodynamic Instability or Need of Rescue Thrombolysis

Hemodynamic deterioration and need of rescue thrombolysis were registered in 27 (6.82%) patients. Demographic, clinical variable and biomarkers were compared between patients that developed hemodynamic instability or required rescue thrombolysis and those without these complications and are revealed in Table 5.

Hs-troponin recorded at admission was significantly higher in patients that developed in hospital hemodynamic instability or required rescue thrombolysis. Echocardiographic parameters in patients with development of hemodynamic instability or need for in hospital thrombolysis are revealed in Table 6.

A significantly lower TAPSE and LVOT VTI was observed in these patients and an increased RV/LV ratio. In multiple regression analysis an LVOT VTI ≤ 15 cm HR = 3.2 (95% CI 1.043–9.810, *p* = 0.042) and RV/LV ≥ 1 HR = 3.54 (95% CI 1.090–11.467, *p* = 0.035) remained independent predictors for hemodynamic deterioration and the need of rescue thrombolysis.

### 3.4. LVOT VTI and RV/LV Ratio in Intermediate-High Risk Patients

**Figure 4:**
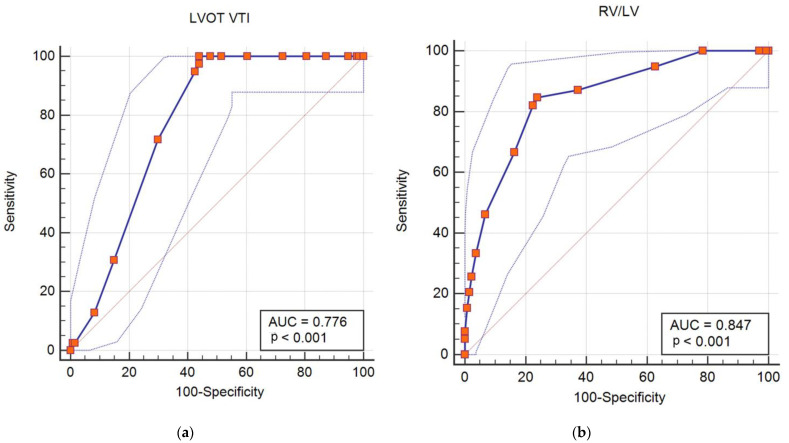
ROC curves for (**a**) LVOT VTI as adverse events predictors in intermediate-high risk PE; (**b**) RV/LV as predictor of adverse events in intermediate-high risk PE.

A number of 173 (43.7%) patients were included in the intermediate-high risk group according to ESC classification. Thirty-nine adverse outcome (78%) were recorded in the intermediate high-risk group. In the subgroup of patients that experienced in hospital adverse outcome 38 (97.44%) had a LVOT VTI ≤ 15 cm and 33 (84.62%) had a RV/LV ratio ≥ 1. A low VTI was recorded in only 44.3% and a RV/LV ratio ≥ 1 in only 23.88% from those without in hospital adverse outcome. In intermediate-high risk patients, LVOT VTI ≤ 15 cm as predictor of adverse in hospital outcome (death, resuscitated cardiac arrest, hemodynamic instability or need of rescue thrombolysis) had a sensitivity of 97.43%, a specificity of 55.97%, a positive predictive value of 39.18% and a negative predictive value of 98.68%. The AUC for LVOT VTI was 0.776 (95% CI 0.696–0.838) (Figure 4a). In intermediate-high risk PE patients, the AUC for RV/LV was 0.847 (95% CI 0.78–0.915) (Figure 4b). RV/LV ≥ 1 as predictor of in hospital adverse outcome had a sensitivity of 84.61%, a specificity of 76.12%, a positive predictive value of 50.77% and a negative predictive value of 94.44%.

**Figure 5:**
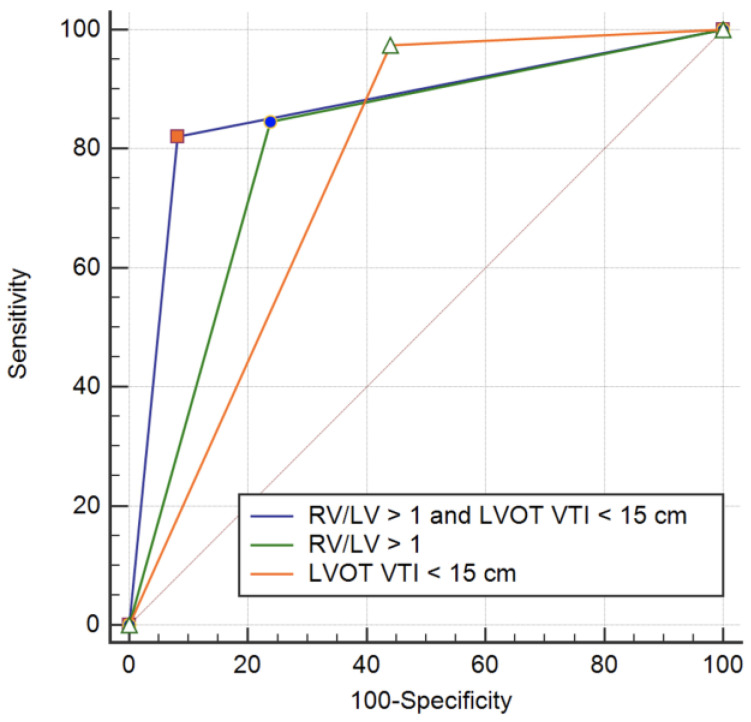
ROC curves for adverse events prediction in intermediate-high risk PE.

The AUC for an LVOT VTI ≤ 15 cm was 0.767 (95% CI 0.696–0.838) and for a RV/LV ≥ 1 was 0.804 (95% CI 0.725–0.882) (Figure 5). The combined criteria: LVOT VTI ≤ 15 mmm and RV/LV ≥ 1 had a sensitivity of 82.051%, a specificity of 94.62%, a positive predictive value of 74.42% and a negative predictive value of 91.79%. The AUC for a combined parameter was 0.869 (95% CI 0.794–0.945) (Figure 5).

In the intermediate low risk PE group, there were 11 adverse events (22%). LVOT VTI ≤ 15 cm was recorded in all the patients with adverse outcome. The sensitivity of a LVOT VTI ≤ 15 cm was 100%, specificity was 99.6%, the positive predictive value was 93.87% and the negative predictive value was 100%.

## 4. Discussion

Echocardiographic parameters correlated with in hospital adverse outcome in patients with PE and normal BP at admission were studied. Main results of this research showed that RV/LV ratio and LVOT VTI were independent predictors of in-hospital adverse outcome. The combination of these two echocardiographic parameters that reflect both RV dysfunction and LV underfilling can reliably identify patients at increased risk of in hospital death, cardiac arrest, hemodynamic deterioration and need for reperfusion therapy. Obstructive shock from acute PE raises pulmonary artery and right ventricular pressure, causing RVD but also a decrease of blood flow across the pulmonary capillary bed to the left heart resulting in LV underfilling and promotes right-to-left septal bowing with a net effect of decreased LV stroke volume. Besides these echocardiographic parameters, several other demographics, clinical risk factors and laboratory parameters were also evaluated regarding their prognostic significance. In univariate analysis previous DVT/PE and hs-troponin were significantly increased in the adverse event group in multivariate analysis only hs troponin remained independent predictor of adverse in hospital outcome. A low BP at admission, female sex and an increased NT-proBNP and hs-troponin were more commonly encountered in the group that experienced cardiac arrest, but these parameters did not persist as independent predictors in a multivariate model.

Risk stratification of hemodynamically stable patients with PE remains a challenge, and although is well known that RVD affects short-term prognosis, the best echocardiographic parameters that can predict adverse outcomes are still an area of debate. The presence of RVD among normotensive patients with PE is a predictor of an increased risk of hemodynamic decompensation [16,19,34]. RVD diagnosed by different echo parameters is also associated with mortality rates from 4.3% to 16.4% [10,13].

An increased RV to LV ratio was found to be correlated with short term adverse outcome including death, cardiac arrest, hemodynamic deterioration and the need of rescue thrombolysis and a ratio ≥ 1 persisted as an independent predictor of in hospital complications after multivariate analysis in our study with a HR of 3.6.

RV/LV is easy to measure and is one of the most used parameters for prognostic assessment in previous studies [16,35]. This was the most important criteria used to identify RVD in the largest study on thrombolysis in intermediate risk pulmonary embolism [36]. RV to LV ratio more than 1 and tricuspid annular plane systolic excursion (TAPSE) less than 16 mm were independent predictors of PE-related mortality and hemodynamic collapse or rescue thrombolysis within the first 30 days in 490 normotensive patients with PE in the study of Pruszczyk et al. An RV/LV ratio ≥ 1 had a sensitivity of 74%, a specificity of 63% and a high negative predictive value of 95%, with a HR of 2.5 (95% CI 1.2–5.7) [37].

Simple measurement of RV/LV diameter ratio by the emergency department specialist on CTPA proved useful in risk stratification of patients with PE in the study of Cho et al. [38]. The RV/LV diameter ratio on transverse CT sections has the strongest predictive value and most robust evidence base for adverse clinical outcomes in patients with acute PE in a systematic review and meta-analysis performed by Meinel et al. [35]. Khemasuwan et al. demonstrated that the ratio of RV to LV end-diastolic diameter with a HR, of 4.4; (95% CI, 1.3–15) and other three parameters: RV systolic pressure, TAPSE and inferior vena cava collapsibility were independently associated with mortality in patients presenting with acute PE who were admitted to the intensive care unit [39].

In the present study, although RV/LV ratio was significantly higher and a RV/LV ratio ≥ 1 was more commonly encountered in the group of patients that experienced death or cardiac arrest, in multivariate analysis RV/LV ratio didn’t t persisted as an independent predictor for short-term combined endpoint of death and resuscitated cardiac arrest, but our study included only patients at intermediate risk.

TAPSE is a quantitative echocardiographic parameter obtained in M-mode that does not require special training for measurement and can provide objective information about RV function. In the present study although TAPSE was correlated in univariate analysis with adverse outcome (including death, cardiac arrest, hemodynamic deterioration, and the need of rescue thrombolysis) its predictive value didn’t t persist in a multivariate model. TAPSE was an independent predictor of survival in normotensive patients with PE in a multicentric prospective study of Lobo et al. and a TAPSE ≤ 16 mm identified those patients with a higher risk of death (HR 4.4; 95% CI 1.3–15.3; *p* = 0.02) [15]. TAPSE was also preferred for risk stratification in normotensive patients with PE [37].

LVOT VTI can estimate cardiac function and is frequently used in the management of critical patients with shock [40,41]. Intensive care and emergency physicians are using more often transthoracic echocardiography for evaluation of patients with hemodynamic instability. Stroke volume is obtained as a product of LVOT cross sectional area by LVOT VTI, but because LVOT cross sectional area is constant any change in stroke volume is dependent on variations of LVOT VTI [30]. On the other hand, due to ellipsoid shape of the LVOT, estimation of the area of LVOT represents the major source of error in calculating cardiac output. Using LVOT VTI alone rather than stroke volume has been suggested for estimation of cardiac output [28].

Although cardiac output can be evaluated with 2D Simpson s method it seems that LVOT VTI is more accurate and can be used as a surrogate for the stroke volume. A value above 18 cm represents an adequate stroke volume, whereas a value ≤ 15 cm defines a reduced left ventricular stroke volume [38]. LVOT VTI is a feasible parameter in various studies. Bergenzaun et al. [42] obtained LVOT VTI in 95% of all examinations in patients with shock and mechanical ventilation whereas other studies revealed a feasibility for LVOT VTI around 78% [42,43]. Regarding reliability of this parameter the reported intra and interobserver variabilities are low, between 3–8% in various studies [42,43,44,45].

A low LVOT VTI (≤15 cm) is associated with worse prognosis in patients with acute PE [27] and in patients with acute decompensated heart failure [28,31]. Ultrasound assessment of patients in shock is becoming the standard of care in emergency and critical care settings worldwide and LVOT VTI is commonly used being part of point-of-care ultrasound protocol [40]. Low LVOT VTI was associated with adverse outcome in patients with PE and proved to have a prognostic value in patients with intermediate risk PE, guiding risk stratification and management in the study of Yuriditsky et al. Fifty-eight percent of intermediate-high risk patients had low VTI. For the entire cohort a low LVOT VTI was associated with in hospital mortality or cardiac arrest HR 6.95% (95% CI 2–17.9, *p* = 0.0014) and shock or need for reperfusion (HR 23.3, 95% CI 6.6–82.1, *p* < 0.0001). Low LVOT VTI was more predictive of shock than death or cardiac arrest [27].

Similar results were observed in our study. LVOT VTI ≤ 15 cm was an independent predictor of in hospital adverse outcome (including in hospital death, cardiac arrest, development of cardiogenic shock, need of rescue thrombolysis). ROC analysis showed an AUC of 0.865, a good accuracy in predicting adverse outcome, a good sensitivity and specificity although positive predictive value remained relatively low. The only independent predictor for death or cardiac arrest in our study as in the study performed by Yuriditsky et al. was a low LVOT VTI. A low LVOT VTI was also an independent predictor of shock or the need or reperfusion in the present study. But in our study a low LVOT VTI was a stronger predictor of death and cardiac arrest than of shock or hemodynamic deterioration, probably because our cohort encompasses only patients at intermediate risk. A low VTI was recorded in 97 patients from 173 included in intermediate high-risk class (56.06%) and this is very similar with the results obtained in the study of Yuriditski et al. [27].

The value of LVOT VTI in predicting adverse outcome was described also in a retrospective study performed by Prosperi-Porta et al. A low stroke volume index was associated with in-hospital death or cardiopulmonary decompensation in normotensive patients with PE. Stroke volume index had excellent performance compared with other clinical and echocardiographic variables. Most often, the authors derived stroke volume index from the LVOT VTI, an echocardiographic surrogate of stroke volume that, as shown in this cohort, can be obtained in the overwhelming majority of patients. The C-statistic for stroke volume index (0.87; 95% CI, 0.78–0.95) outperformed every other marker of RVD including the well-studied TAPSE and the Bova staging system. The ability of stroke volume index to discriminate the prognosis of patients with acute PE was preserved even after comparisons against multiple other RVD markers and after sensitivity analyses [46].

Another recent study on patients with intermediate risk PE confirmed that LVOT VTI (HR 4.212, 5–95% CI 1.384–12.820, *p* = 0.011), as well as stroke volume index (HR 11.199, 5–95% CI 2.697–48.096, *p* = 0.001), were independent predictors of short-term mortality as in our study. Also, other echocardiographic parameters as: right atrial enlargement (HR 3.432, 5–95% CI 1.193–9.876, *p* = 0.022) and the ratio between tricuspid annulus plane excursion and pulmonary arterial systolic pressure (TAPSE/PASP) (HR 4.833, 5–95% 1.230–18.986, *p* = 0.024) were found to have predictive value in the study of Falsetti et al. In this study as in our study neither RV/LV nor troponin I resulted associated with impaired survival [47].

In the intermediate high-risk group were registered 78% of the in hospital adverse outcomes. Most patients (97.44%) that reached the composite outcome of cardiac arrest, shock or need of reperfusion had a VTI ≤ 15 cm and only 44.3% of patients without adverse outcome during hospitalization had a low LVOT VTI. Similar results were obtained in the study of Yuriditski et al. where 92.3% of patients with adverse outcome had a low LVOT VTI and only 42.6% of outcome-negative patients had a VTI ≤ 15 cm [27].

RVD is correlated with an elevated risk of short-term mortality, but the positive predictive value of various echocardiographic parameters is low (<10%) [22]. However, it is generally accepted that detection of RVD at echocardiography can identify patients with high-risk of hemodynamic deterioration despite anticoagulation. Combining more echocardiographic parameters we can increase the positive predictive value. We postulate that concomitant evaluation of the two echocardiographic parameters that showed independent predictive value for risk estimation in the present study and can estimate simultaneously the right and the left heart could increase the positive predictive value of the echocardiographic parameters. The concomitant presence of an LVOT VTI ≤ 15 cm and of a RV/LV ratio ≥ 1 was associated with an increased specificity and with a higher positive predictive value compared to using LVOT VTI or RV/LV ratio alone.

The first study that used evaluation of left and right heart hemodynamic was the study of Kamran et al. Simultaneous information on both right and left heart performance was provided by the ratio pulmonary systolic arterial pressure/left ventricular stroke volume (PSAP/LVSV) an echocardiographic variable that evaluates the interventricular dependence in PE. The authors found that a ratio ≥1 mmHg/mL was associated with an increased risk of adverse short-term outcomes in patients with acute intermediate risk PE [48].

A recent retrospective study that evaluated the relationship of different echocardiographic parameters with treatment strategy in sub massive and massive PE observed that changes in LVOT VTI, rather than echocardiographic markers of right ventricular dysfunction, may be considered a more useful prognostic marker of both dysfunction and improvement after reperfusion therapy [48]. The value of a low LVOT VTI in predicting adverse outcome in patients with intermediate risk PE is because this parameter reflects a reduced stroke volume and probably a subclinical shock, despite a normal BP.

Hemodynamic instability at admission represents a high risk and in this clinical setting immediate reperfusion therapy is mandatory. For hemodynamically stable patients at presentation, further risk stratification of PE is recommended, as it has implications for determining the appropriate therapeutic management approach. The study included intermediate risk patients because further risk stratification in this group and identification of patients at highest risk of poor outcome that will require escalation of therapy remains a challenge. For patients clinically classified at low risk two recent meta-analysis reported that RVD affects prognosis [14,34]. Echocardiography should be considered in the low-risk group to identify those patients that will require hospitalization. In the study performed by Yuriditski et al., most patients (*n* = 109, 58%) were categorized as having intermediate-risk. But the study also included a low number of patients at high risk, respectively (11, 5.9%) with a low LVOT VTI being demonstrated in most of them (10, 90.9%). Low risk patients were better represented in this study (*n* = 68, 36.2%) and low LVOT VTI was present in only in four of them (5.88%) [27]. In the study performed by Kamran et al., that evaluated concomitantly left and right heart hemodynamic, patients with low-risk PE and high-risk (massive) PE were underrepresented and so the performance of pulmonary artery systolic pressure/left ventricle stroke volume (PASP/LVSV) in these patient groups could not be evaluated [48]. The study performed by Antoine et al. had the objective to compare ultrasound-accelerated thrombolysis versus systemic thrombolysis versus anticoagulation alone and their effect on LVOT VTI. This was a retrospective cohort study of subjects with a diagnosis of sub massive or massive pulmonary embolism according to American Heart Association guideline definitions. It was difficult to control for disease severity but in this study most patients had massive-high risk PE. Greater improvement in LVOT VTI was observed in patients treated with ultrasound-accelerated thrombolysis as compared to anticoagulation alone. Changes in LVOT VTI rather than echocardiographic markers of right ventricular dysfunction, were found to be more useful prognostic marker of both dysfunction and improvement after reperfusion therapy [49]. How to better identify patients at risk of hemodynamic deterioration or death in initially stable intermediate risk patients remains a challenge. Both an increased RV to LV ratio and/or a low LVOT VTI are useful for identifying normotensive patients with PE at risk for short term adverse outcome. The combined criteria of a low LVOT VTI ≤ 15 cm and increased RV/LV ratio ≥ 1 had a 75% positive predictive value for in-hospital adverse outcome. The clinical implication is that 75% of those patients that had both criteria developed an adverse outcome during hospitalization. Evaluation of these parameters with point of care ultrasound allows a rapid risk stratification of PE patients. Admission in the intensive care unit and close monitoring is recommended for these patients because they will probably require escalation of therapy.

The limitation of the study is his retrospective nature that may bias the tests performance statistics since echocardiography was performed faster after admission and more complete echocardiographic examinations were recorded in more severe patients. Future prospective studies that will examine the value of combining these two echocardiographic criteria are needed to validate these findings.

## 5. Conclusions

Low LVOT VTI and an increased RV/LV ratio are useful for identifying normotensive patients with PE at risk for short term adverse outcomes. Combining an LVOT VTI ≤ 15 cm with a RV/LV ratio ≥ 1, can identify PE patients with impending risk of clinical deterioration, that require close monitoring with increased specificity and positive predictive value. These two echocardiographic parameters proved good accuracy for risk stratification of patients with intermediate risk PE.

## Figures and Tables

**Table 1:** Comparative baseline demographic, clinical and laboratory parameters in patients with and without in hospital adverse outcomes.

Death, Resuscitated Cardiac Arrest, Hemodynamic Instability and Need of Reperfusion Therapy				*p*
Adverse Outcome	Total (396)	Yes (50)	No (346)	
Age (years)	73.58 ± 11.221	71.22 ± 13.27	73.92 ± 10.87	0.09
Sex (F)	202/396 (51%)	27/50 (54%)	175/346 (50.57%)	0.120
Smoking	134/396 (33.83%)	15/50 (30%)	119/346 (34.39%)	0.26
History of cancer	67/396 (16.92%)	8/50 (16%)	59/346 (17.05%)	0.628
History of cardiovascular disease	302/396 (76.3%)	31/50 (62%)	271/346 (78.32%)	0.01 *
Heart failure	132/396 (33.33%)	14/50 (28%)	118/346 (34.10%)	0.19
Coronary artery disease	86/396 (21.71%)	8/50 (16%)	78/346 (22.54%)	0.14
Valvular heart disease	114/396 (28.79%)	10/50 (20%)	104/346 (30.06%)	0.07
Hypertension	210/396 (53.03%)	20/50 (40%)	190/346 (54.91%)	0.02 *
History of pulmonary disease	106/396 (26.8%)	13/50 (26%)	93/346 (26.88%)	0.896
Obstructive pulmonary disease	79/396 (19.94%)	10/50 (20%)	69/346 (19.94%)	0.49
Restrictive pulmonary disease	19/396 (4.80%)	4/50 (8%)	15/346 (4.33%)	0.12
Combined obstructive and restrictive pulmonary disease	8/396 (2.02%)	2/50 (4%)	6/346 (1.73%)	0.14
Diabetes	82/396 (20.7%)	13/50 (26%)	69/346 (19.94%)	0.324
History of stroke	41/396 (10.35%)	5/50 (10%)	36/346 (10.40%)	0.46
History of recent surgery	23/396 (5.80%)	3/50 (6%)	20/346 (5.78%)	0.47
History of recent orthopedic surgery or major trauma	20/396 (5.05%)	4/50 (8%)	16/346 (4.62%)	0.15
Previous DVT/PE	54/396 (13.63%)	10/50 (20%)	44/346 (12.72%)	0.03 *
BMI (kg/m²)	30.25 ± 6.73	32.87 ± 6.06	27.62 ± 6.69	0.12
Systolic BP (mm Hg)	126.85 ± 22.234	121.73 ± 21.94	128.25 ± 22.17	0.07
HR	91.571 ± 19.14	94.142 ± 19.86	89 ± 18.42	0.368
DVT	69/396 (17.42%)	8/50 (16%)	61/346 (17.63%)	0.649
Cholesterol (mg/dL)	232.6 ± 66.61	251.40 ± 32.88	213.8 ± 89.55	0.06
NT-proBNP (pg/mL)	5267.354 ± 6991.84	7307.27 ± 9350.97	4975 ± 6583.97	0.187
Hs-Troponin (pg/mL)	306.66 ± 579.82	711.054 ± 596.66	193.32 ± 523.62	<0.001 *

Legend: BP—blood pressure, HR—heart rate, DVT—deep vein thrombosis, PE—pulmonary embolism, BMI—body mass index, NT-proBNP—N-terminal pro b-type natriuretic peptide, Hs-Troponin—highly sensitive troponin; * statistically significant (<0.05).

**Table 2:** Comparative baseline echocardiographic parameters in patients with and without in-hospital adverse outcomes.

Death, Resuscitated Cardiac Arrest, Hemodynamic Instability and Need of Reperfusion Therapy				*p*
Adverse Outcome	Total (396)	Yes (50)	No (346)	
TAPSE < 16 mm	116/396 (29.29%)	23/50 (46%)	93/346 (26.88%)	0.022 *
TAPSE (mm)	20.35 ± 6.52	17.68 ± 5.93	20.76 ± 6.51	0.001 *
Paradoxical movement or flattening of IVS	113/396 (28.54%)	20/50 (40%)	93/346 (26.88%)	0.057
PSAP (mmHg)	40.38 ± 12.202	43.66 ± 14.52	39.99 ± 11.808	0.129
Mc Connell sign	124/396 (31.31%)	19/50 (38%)	105/346 (30.34%)	0.283
RV/LV ≥ 1	123/396 (31.1%)	36/50 (72%)	87/346 (25.14%)	<0.001 *
RV/LV	0.93 ± 0.31	1.28 ± 0.45	0.884 ± 0.26	<0.001 *
LVOT VTI (cm)	18.87 ± 4.38	13.88 ± 2.58	19.60 ± 4.10	<0.001 *
LVOT VTI ≤ 15 cm	99/396 (25%)	38/50 (76%)	61/346 (17.63%)	<0.001 *
LVEF (%)	47.60 ± 8.13	48.25 ± 8.86	47.51 ± 8.06	0.610
IVC (mm)	21.94 ± 8.07	23.18 ± 7.97	20.25 ± 8.44	0.451

Legend: TAPSE—tricuspid annular plane systolic excursion, IVS—interventricular septum, PSAP—pulmonary systolic arterial pressure, RV—right ventricle, LV—left ventricle, LVOT VTI—left ventricle outflow tract velocity-time integral, LVEF—left ventricle ejection fraction, IVC—inferior vena cava; * statistically significant (<0.05).

**Table 3:** Comparative baseline demographic, clinical and laboratory parameters in patients with and without in hospital death or resuscitated cardiac arrest.

Death or Resuscitated Cardiac Arrest	Yes (23)	No (373)	*p*
Age (years)	73.00 ± 15.97	73.61 ± 10.890	0.799
Sex (F)	13/23 (56.52%)	189/373 (50.67%)	0.001 *
Smoking	7/23 (30.43%)	127/373 (34%)	0.36
History of cancer	4/23 (17.39%)	63/373 (16.89%)	0.928
History of cardiovascular disease	14/23 (60.87%)	288/373 (77.21%)	0.797
Heart failure	8/23 (34.78%)	124/373 (33.24%)	0.43
Coronary artery disease	5/23 (21.74%)	81/373 (21.72%)	0.49
Valvular heart disease	6/23 (26.08%)	108/373 (28.95%)	0.38
Hypertension	11/23 (47.82%)	199/373 (53.35%)	0.3
History of pulmonary disease	5/23 (21.74%)	101/373 (27.08%)	0.676
Obstructive pulmonary disease	3/23 (13.04%)	76/373 (20.38%)	0.41
Restrictive pulmonary disease	1/23 (4.35%)	18/373 (4.83%)	0.45
Combined obstructive and restrictive pulmonary disease	1/23 (4.35%)	7/373 (1.88%)	0.20
Diabetes	6/23 (26.09%)	76/373 (20.38%)	0.513
History of stroke	4/23 (17.39%)	37/373 (9.92%)	0.12
History of recent surgery	1/23 (4.34%)	22/373 (5.90%)	0.37
History of recent orthopedic surgery or major trauma	2/23 (8.69%)	18/373 (4.83%)	0.20
Previous DVT/PE	4/23 (17.39%)	50/373 (13.40%)	0.13
BMI (kg/m²)	33.00 ± 5.87	28.60 ± 6.96	0.20
Systolic BP (mmHg)	115.83 ± 16.88	128.18 ± 22.4	0.002 *
HR	96.2 ± 10.59	87.5 ± 16.46	0.521
DVT	1/23 (4.34%)	68/373 (18.23%)	0.08/0.007 *
Cholesterol (mg/dL)	249 ± 45.17	225.57 ± 76.05	0.56
NT-proBNP (pg/mL)	11,029.0 ± 11,349.29	4883.24 ± 6488.53	0.01 *
Hs-Troponin (pg/mL)	814.70 ± 718.55	238.475 ± 525.151	<0.001 *

Legend: BP—blood pressure, HR—heart rate, DVT—deep vein thrombosis, PE—pulmonary embolism, BMI—body mass index, NT-proBNP—N-terminal pro b-type natriuretic peptide, Hs-Troponin—high sensitive troponin; * statistically significant (<0.05).

**Table 4:** Comparative baseline echocardiographic parameters in patients with and without in hospital death or resuscitated cardiac arrest.

Death or Resuscitated Cardiac Arrest	Yes (23)	No (373)	*p*
TAPSE < 16 mm	9/23 (39.13%)	107/373 (28.68%)	0.431
TAPSE (mm)	19.17 ± 6.27	20.43 ± 6.53	0.36
Paradoxical movement or flattening of IVS	11/23 (47.82%)	102/373 (27.34%)	0.036 *
PSAP (mmHg)	45.94 ± 16.213	39.98 ± 11.808	0.059
McConnel sign	10/23 (43.47%)	114/373 (30.56%)	0.07
RV/LV ≥ 1	15/23 (65.22%)	108/373 (28.95%)	0.001 *
RV/LV	1.2 ± 0.44	0.92 ± 0.303	<0.001 *
LVOT VTI (cm)	13.65 ± 2.75	19.19 ± 4.25	<0.001 *
LVOT VTI ≤ 15 cm	20/23 (86.96%)	79/373 (21.18%)	<0.001 *
LVEF (%)	47.93 ± 11.472	47.59 ± 7.96	0.879
IVC (mm)	24.12 ± 6.75	20.36 ± 8.90	0.33

Legend: TAPSE—tricuspid annular plane systolic excursion, IVS—interventricular septum, PSAP—pulmonary systolic arterial pressure, RV—right ventricle, LV—left ventricle, LVOT VTI—left ventricle outflow tract velocity-time integral, LVEF—left ventricle ejection fraction, IVC—inferior vena cava; * statistically significant (<0.05).

**Table 5:** Comparative baseline demographic, clinical and laboratory parameters in patients with and without in hospital hemodynamic instability or need of reperfusion therapy.

Hemodynamic Instability or Reperfusion Therapy	Yes (27)	No (369)	*p*
Age (years)	69.70 ± 10.550	73.86 ± 11.229	0.064
Sex (F)	13/27 (48.15%)	189/369 (51.22%)	0.618
Smoking	8/279 (9.63%)	126/369 (34.14%)	0.31
History of cancer	4/27 (14.81%)	63/369 (17.07%)	0.463
History of cardiovascular disease	17/27 (62.96%)	285/369 (77.24%)	0.095
Heart failure	6/27 (22.22%)	126/369 (31.81%)	0.22
Coronary artery disease	3/27 (11.11%)	83/369 (22.49%)	0.14
Valvular heart disease	4/27 (14.81%)	110/369 (29.81%)	0.10
Hypertension	9/27 (33.33%)	201/369 (54.47%)	0.08
History of pulmonary disease	8/27 (29.63%)	98/369 (26.56%)	0.729
Obstructive pulmonary disease	5/27 (18.52%)	74/369 (20.05%)	0.42
Restrictive pulmonary disease	2/27 (7.41%)	17/369 (4.61%)	0.27
Combined obstructive and restrictive pulmonary disease	1/27 (3.70%)	7/369 (1.89%)	0.25
Diabetes	7/27 (25.93%)	75/369 (20.33%)	0.489
History of stroke	1/27 (3.70%)	40/369 (10.84%)	0.12
History of recent surgery	2/27 (7.41%)	21/369 (5.69%)	0.35
History of recent orthopedic surgery or major trauma	2/27 (7.41%)	18/369 (4.87%)	0.28
Previous DVT/PE	6/27 (22.22%)	48/369 (13%)	0.08
BMI (kg/m²)	255 ± 14.14	227 ± 74.14	0.34
Systolic BP (mm Hg)	127.69 ± 24.382	126.74 ± 22.006	0.837
HR	96.2 ± 10.59	87.5 ± 16.46	0.521
DVT	7/27 (25.93%)	62/369(16.80%)	0.19
Cholesterol (mg/dL)	255 ± 15.14	227 ± 74.14	0.15
NT-proBNP (pg/mL)	3585 ± 5083	5377 ± 7102	0.459
Hs-Troponin (pg/mL)	588.02 ± 400.018	272. 67 ± 590.27	0.007 *

Legend: BP—blood pressure, HR—heart rate, DVT—deep vein thrombosis, PE—pulmonary embolism, BMI—body mass index, NT-proBNP—N-terminal pro b-type natriuretic peptide, Hs-troponin—high sensitive troponin; * statistically significant (<0.05).

**Table 6:** Comparative baseline echocardiographic parameters in patients with and without in hospital hemodynamic instability or need of reperfusion therapy.

Hemodynamic Instability or Reperfusion Therapy	Yes (27)	No (369)	*p*
TAPSE < 16 mm	14/27 (51.85%)	102/369 (27.64%)	0.013 *
TAPSE (mm)	16.40 ± 5.43	20.66 ± 6.50	0.001 *
Paradoxical movement or flattening of IVS	9/27 (33.33%)	104/369 (28.18%)	0.57
PSAP (mm Hg)	40.85 ± 12.164	40.35 ± 12.23	0.889
Mc Connell sign	9/27 (33.33%)	115/369 (31.17%)	0.82
RV/LV ≥ 1	22/27 (81.48%)	101/369 (27.37%)	<0.001 *
RV/LV	1.34 ± 0.45	0.90 ± 0.29	<0.001 *
LVOT VTI (cm)	13.96 ± 2.38	19.23 ± 4.27	<0.001 *
LVOT VTI ≤ 15 cm	18/27 (66.66%)	81/369 (21.95%)	<0.001 *
LVEF (%)	48.45 ± 7.015	47.54 ± 8.21	0.612
IVC (mm)	20.66 ± 12.05	22.19 ± 7.65	0.77

Legend: TAPSE—tricuspid annular plane systolic excursion, IVS—interventricular septum, PSAP—pulmonary systolic arterial pressure, RV—right ventricle, LV—left ventricle, LVOT VTI—left ventricle outflow tract velocity-time integral, LVEF—left ventricle ejection fraction, IVC—inferior vena cava; * statistically significant (<0.05).

## Data Availability

Data are available in the electronic registration data base of the Clinical County Emergency Hospital of Oradea, Bihor County, Romania.
